# Winter rye as a bioenergy feedstock: impact of crop maturity on composition, biological solubilization and potential revenue

**DOI:** 10.1186/s13068-015-0225-z

**Published:** 2015-02-27

**Authors:** Xiongjun Shao, Kay DiMarco, Tom L Richard, Lee R Lynd

**Affiliations:** Thayer School of Engineering at the Dartmouth College, 14 Engineering Drive, Hanover, NH 03755 USA; DOE BioEnergy Science Center, Oak Ridge National Laboratory, Oak Ridge, TN 37831 USA; Penn State University, 225 Agricultural Engineering Building, University Park, PA 16802 USA; Enchi Corporation, Lebanon, NH 03766 USA

**Keywords:** Winter rye, *Secale cereale L*, Unpretreated, Lignocellulosic biomass, Growth stage, Boot stage, Harvest maturity, Biological solubilization, Carbohydrate solubilization, Protein recovery, Consolidated bioprocessing, SSCF, *Clostridium thermocellum*

## Abstract

**Background:**

Winter annual crops such as winter rye (*Secale cereale L*) can produce biomass feedstock on seasonally fallow land that continues to provide high-value food and feed from summer annuals such as corn and soybeans. As energy double crops, winter grasses are likely to be harvested while still immature and thus structurally different from the fully senesced plant material typically used for biofuels. This study investigates the dynamic trends in biomass yield, composition, and biological solubilization over the course of a spring harvest season.

**Results:**

The water soluble fraction decreased with increasing maturity while total carbohydrate content stayed roughly constant at about 65%. The protein mass fraction decreased with increasing maturity, but was counterbalanced by increasing harvest yield resulting in similar total protein across harvest dates. Winter rye was ground and autoclaved then fermented at 15 g/L total solids by either (1) *Clostridium thermocellum* or (2) simultaneous saccharification and cofermentation (SSCF) using commercial cellulases (CTec2 and HTec2) and a xylose-fermenting *Saccharomyces cerevisiae* strain. Solubilization of total carbohydrate dropped significantly as winter rye matured for both *C. thermocellum* (from approximately 80% to approximately 50%) and SSCF (from approximately 60% to approximately 30%). *C. thermocellum* achieved total solubilization 33% higher than that of SSCF for the earliest harvest date and 50% higher for the latest harvest date. Potential revenue from protein and bioethanol was stable over a range of different harvest dates, with most of the revenue due to ethanol. In a crop rotation with soybean, recovery of the soluble protein from winter rye could increase per hectare protein production by 20 to 35%.

**Conclusions:**

Double-cropping winter rye can produce significant biomass for biofuel production and feed protein as coproduct without competing with the main summer crop. During a 24-day harvest window, the total carbohydrate content remained relatively constant while the early-harvest yielded much higher carbohydrate solubilization for both *C. thermocellum* fermentation and SSCF. *C. thermocellum* fermentation achieved higher carbohydrate solubilization than SSCF across all growth stages tested. Although winter rye’s yield, composition, and biological reactivity change rapidly in the spring, it offers a substantial and stable income across the harvest season and thus flexibility for the farmer.

**Electronic supplementary material:**

The online version of this article (doi:10.1186/s13068-015-0225-z) contains supplementary material, which is available to authorized users.

## Background

Lignocellulosic biomass is of interest for sustainable production of fuels and chemicals [1]. Winter cover crops could be good feedstocks because their production uses readily available farm equipment and techniques, there is little or no competition with food crops [2], they can positively impact soil and water quality [3-5], and these winter crops offer important feed protein coproduct opportunities [5]. In much of the United States corn belt cover crops are commonly established in September or October and harvested or plowed under in late April or early May, so they do not interfere with summer annuals like corn or soybeans. This ‘off-season’ production offers potential synergies rather than competition with food crops and is thus an attractive way to integrate bioenergy crops with traditional agricultural systems. Because these are annual crops, they have a lower initial investment than perennial bioenergy crops and may be more attractive to farmers concerned about long-term biomass markets during the early stages of biofuel industrial development. Spring-harvested winter crops will contain significant protein nitrogen and other plant and animal nutrients. Recovery of such nutrients for animal feed is a potential added source of revenue and may offer life cycle benefits compared to alternative modes of feed production.

Among the variety of legume and non-legume plants commonly used as winter crops, winter rye (*Secale cereale L*) shows the highest yield potential in the temperate corn belt including Pennsylvania [6]. Winter rye is an annual grain crop that has been traditionally grown as animal feed as well as for flour and beverages, and is today primarily grown as a cover crop for soil and water conservation. Growing winter rye as a second crop on land also used to grow corn and soy could produce 150 million dry tons per year in the USA [2], which has a liquid fuel potential comparable to that of the current US ethanol industry. In many temperate agricultural regions, winter rye and other winter grains would be harvested before reaching full maturity. These immature grasses are structurally different than the fully senesced plant material typically used for biochemical conversion to biofuel and have long been known to be much more digestible than mature grasses by both livestock farmers and ruminant nutritionists [7]. Among many considerations, evaluating winter rye as a biofuel feedstock thus requires an understanding of the impact of plant maturity on biochemical processes. Sun and Chen [8] measured the sugar yield of winter rye straw after various sulfuric acid pretreatment severities followed by enzymatic hydrolysis with cellulases. A maximum of 197.1 mg of total reducing sugars per gram of dry matter was reported. They found the major compositional components to be glucan (33.12%), acid insoluble lignin (19.80%), xylan (10.46%), Ash (6.15%), and arabinan (2.47%). However, senesced winter rye straw remaining after grain harvest is more mature than winter rye grown as a lignocellulosic double crop and harvested before or at flowering.

Although there are several growth stage scales that describe the maturity of cereal grasses, they have similar physiological descriptions and terms. Starting with ‘germination’, there are then several stages of ‘seedling growth’ followed by ‘tillering’, which is the addition of leaves to the main shoot; ‘stem elongation’; ‘booting’, where the seed head is detectable inside of the stem; ‘heading’, which is when the seed head becomes visible, also called ‘inflorescence emergence’; ‘flowering’, also called ‘anthesis’; and lastly, ‘grain development’, an example of which is ‘soft dough’ and death or dormancy. In the Northeast, winter rye grown as a cover or second crop will rarely make it to flowering by early May when farmers are eager to plant their summer crops. The compositional difference between young and mature plants is likely to be a factor when estimating fuel yield and costs. As the growing season progresses, yield is increased but the feedstock’s amenability to biochemical conversion processes may be decreased. Decades of research into the use of cereal grasses for animal forage have clearly shown a decrease in digestibility as plants mature [7].

Several prior studies have investigated the changes in winter rye composition as a function of maturity. Kantar et al. [9] sampled weekly from tillering to dough development and characterized each stage in terms of crude protein (CP), neutral detergent fiber (NDF), and *in vitro* true digestibility (IVTD). In biomass energy terms, NDF can be described an undifferentiated combination of cellulose, hemicellulose, and lignin while IVTD is a measure of digestibility in a buffer/rumen fluid mixture. Fisher and Fowler [10] started sampling at late boot stage in 10-day intervals till maturity and analyzed for CP, digestible organic matter (DOM), NDF, and acid detergent fiber (ADF). The hemicellulose can be estimated as the difference between NDF and ADF, but lignin was not measured so percent cellulose cannot be deduced. Helsel and Thomas [11] include lignin but analyzed the later growth stages of heading, milk, and soft dough-growth stages. All of the studies discussed above showed decreased digestibility with plant maturity and several point to boot stage as a physiological indicator of the optimal time to harvest for high digestibility balanced with a decent yield for ruminant feed applications. Harvest stage trials on rye and other small grains used as whole crops for forage show that the tradeoff for boot stage harvesting is a 30% to 60% reduction in yield compared to soft dough stage [12]. Harvesting a winter grain energy double-crop at the soft dough state could be an option in the southern regions of the corn belt, but the greater yield may still have a tradeoff with digestibility in the context of an industrial biorefinery.

Various process configurations have been proposed for biological processing of lignocellulosic biomass to produce fuels and chemicals, including separate hydrolysis and fermentation (SHF), simultaneous saccharification and fermentation (SSF), simultaneous saccharification and cofermentation (SSCF), and consolidated bioprocessing (CBP) [13]. Fungal cellulase hydrolysis combined with yeast fermentation is a prominent model system for SHF, SSF, and SSCF. *Clostridium thermocellum* fermentation, potentially in co-culture with a companion 5-carbon sugar utilization strain, is a prominent model system for CBP. Commercial deployment of *C. thermocellum* or other thermophiles offers a great potential for cost reduction by eliminating costly cellulase addition while consolidating capital equipment. Additional improvements on yield and titer are necessary for commercial application, although genetic tools have already been successfully applied to increase ethanol yield of *C. thermocellum* and *T. saccharolyticum* fermentations [14,15].

In the lignocellulosic biofuel field, the focus of most studies has been woody feedstocks or senescent grass harvested at the end of the season. For such feedstocks, hydrolysis yields using industry standard fungal cellulases are generally low (e.g., ≤20%) so some form of pretreatment is thought to be required in order to achieve the high hydrolysis yields necessary for commercial viability. After an intensive (and expensive) pretreatment process, lignocellulose conversion via enzymatic hydrolysis using fungal cellulases typically achieves about 70% to 90% cellulose hydrolysis yields with on the order of 5 days required for hydrolysis and fermentation [16,17]. Grass forage, by contrast, is usually harvested or grazed while immature at intervals of a few weeks. When fed to livestock, the grass enters the ruminant stomach without any pretreatment other than mastication, and cellulose solubilization of 60% to 80% is typically achieved in the rumen in about 24 h [18]. We hypothesize that the enzymes and microorganisms used in SSCF and CBP can mimic the rumen microbial ecosystem and digest a high percentage of the carbohydrates found in immature plants without conventional pretreatment.

This study was undertaken with the objectives of advancing the understanding of dynamic trends over the spring harvest season with respect to plant maturity and biomass characteristics, the impact of these trends on biological conversion and potential products and revenue, and comparing the relative effectiveness of fungal cellulase and *C. thermocellum* fermentation at mediating solubilization. In order to be relevant to double cropping with corn and soybeans in the Northeastern United States, our study starts with younger growth stages than much of the previous work and includes measurements for lignin since it has been demined to be highly negatively correlated with digestibility [7,19].

## Results and discussion

### Crop maturity and yield

Winter rye samples were collected at four different times during the spring of 2012, starting with April 16 and ending on May 10 which is the recommended planting date for corn in Pennsylvania. On April 16, the non-fertilized plots displayed a mix of stem elongation and booting but the fertilized plots consisted of plants in the stages of late booting and inflorescence (seed head emergence). On May 4, approximately 30% of plants in the fertilized plots had started to flower and continued to flower until the last harvest date of May 10, by which time the majority of the non-fertilized plants were beginning to flower. Nitrogen deficiency is known to delay the reproductive phonological development of plants [20,21]. The observed differences in plant maturation rates between fertilizer treatments indicate a nitrogen deficiency in the soil that likely impacted the protein content reported.

For the paired fertilizer treatments, plants without nitrogen fertilizer were less mature during the early harvests (Table 1) and consistently had lower biomass yield (*P* value = 0.002) than those fertilized with 60 kg per hectare N. Biomass yield increased until around the third harvesting date (May 4, 2012) when the seed heads emerged, and then plateaued without much change for the fourth harvesting date which occurred at the time of flowering.

**Table 1:** **Production data for winter rye samples**

**Harvesting date**	**Fertilization (kg N/ha)**	**Sample ID designation**	**Maturity**	**Biomass yield (tons/ha)**
April 16, 2012	0	April 16-0 N	Stem elongation, booting	3.92 ± 0.29
April 16, 2012	60	April 16-60 N	Late booting, start of heading	6.28 ± 0.68
April 27, 2012	0 (wilted on field)	April 27-0 N-wilted	Late booting, start of heading	5.41 ± 0.60
April 27, 2012	0	April 27-0 N	Late booting, start of heading	5.41 ± 0.60
April 27, 2012	60	April 27-60 N	Heading	6.84 ± 0.87
May 4, 2012	0	May 4-0 N	Heading	5.63 ± 0.46
May 4, 2012	60	May 4-60 N	Heading and start of Flowering	8.46 ± 0.99
May 10, 2012	0	May 10-0 N	Flowering	5.89 ± 1.52
May 10, 2012	60	May 10-60 N	Flowering	8.50 ± 0.58

### Feedstock characterization

Samples from each winter rye sampling event (Table 1) were analyzed with respect to water solubility and composition, with the results shown in Table 2. Detailed composition of the water soluble and insoluble fractions is provided in supplemental Additional file 1: Tables S1 and S2. The water soluble fraction decreased as the winter rye matured, dropping from over 30% to around 15% in 25 days. Soluble carbohydrate content decreased with the soluble fraction but insoluble carbohydrates increased as a fraction of overall mass, making the total carbohydrate content relatively stable at around 65% for all harvesting dates. Acetyl groups as a fraction of dry weight were about constant. Total protein as a fraction of total mass and water soluble protein as a fraction of total protein both decreased for later harvesting dates, and the lignin fraction of total mass increased as the plants matured (Table 2).

**Table 2:** **Composition of winter rye samples on a dry matter (DM) basis**

**Sample ID**	**Soluble fraction of DM**	**Carbohydrate**	**Carbohydrate**		**Acetyl**	**Protein**	**Protein**		**Lignin**	**Ash**	**Total**	**Unknown**	
			**Insoluble**	**Soluble**			**Insoluble**	**Soluble**				**Insoluble**	**Soluble**
April 16-0 N	36.4%	*64.8%*	41.4%	23.4%	*2.2%*	*8.7%*	1.2%	7.5%	*9.6%*	*0.5%*	85.8%	8.6%	5.6%
April 16-60 N	32.8%	*63.6%*	44.1%	19.6%	*2.4%*	*8.8%*	1.3%	7.5%	*10.2%*	*0.5%*	85.6%	8.7%	5.7%
April 27-0 N-wilted	30.0%	*65.5%*	44.0%	21.4%	*2.4%*	*6.5%*	1.3%	5.2%	*12.0%*	*0.8%*	87.2%	9.4%	3.4%
April 27-0 N	28.6%	*67.0%*	47.4%	19.6%	*2.6%*	*6.6%*	1.7%	4.8%	*12.3%*	*0.6%*	89.0%	6.8%	4.2%
April 27-60 N	27.9%	*64.7%*	47.0%	17.7%	*2.6%*	*6.9%*	2.0%	4.9%	*12.2%*	*0.6%*	86.9%	7.8%	5.3%
May 4-0 N	19.3%	*64.8%*	53.6%	11.2%	*3.0%*	*6.0%*	1.8%	4.2%	*14.3%*	*0.6%*	88.7%	7.5%	3.9%
May 4-60 N	20.6%	*64.4%*	52.2%	12.2%	*2.7%*	*6.5%*	2.3%	4.2%	*14.2%*	*0.6%*	88.4%	7.3%	4.3%
May 10-0 N	15.2%	*65.4%*	56.3%	9.1%	*3.0%*	*4.6%*	1.9%	2.6%	*15.6%*	*0.7%*	89.2%	7.4%	3.4%
May 10-60 N	16.5%	*65.3%*	55.0%	10.3%	*2.8%*	*5.3%*	2.6%	2.7%	*16.0%*	*0.6%*	90.1%	6.5%	3.4%

Note: the values in ‘Total’ are the sum of the italic values.

### Carbohydrate conversion by *C. thermocellum* compared to SSCF

**Figure 1:**
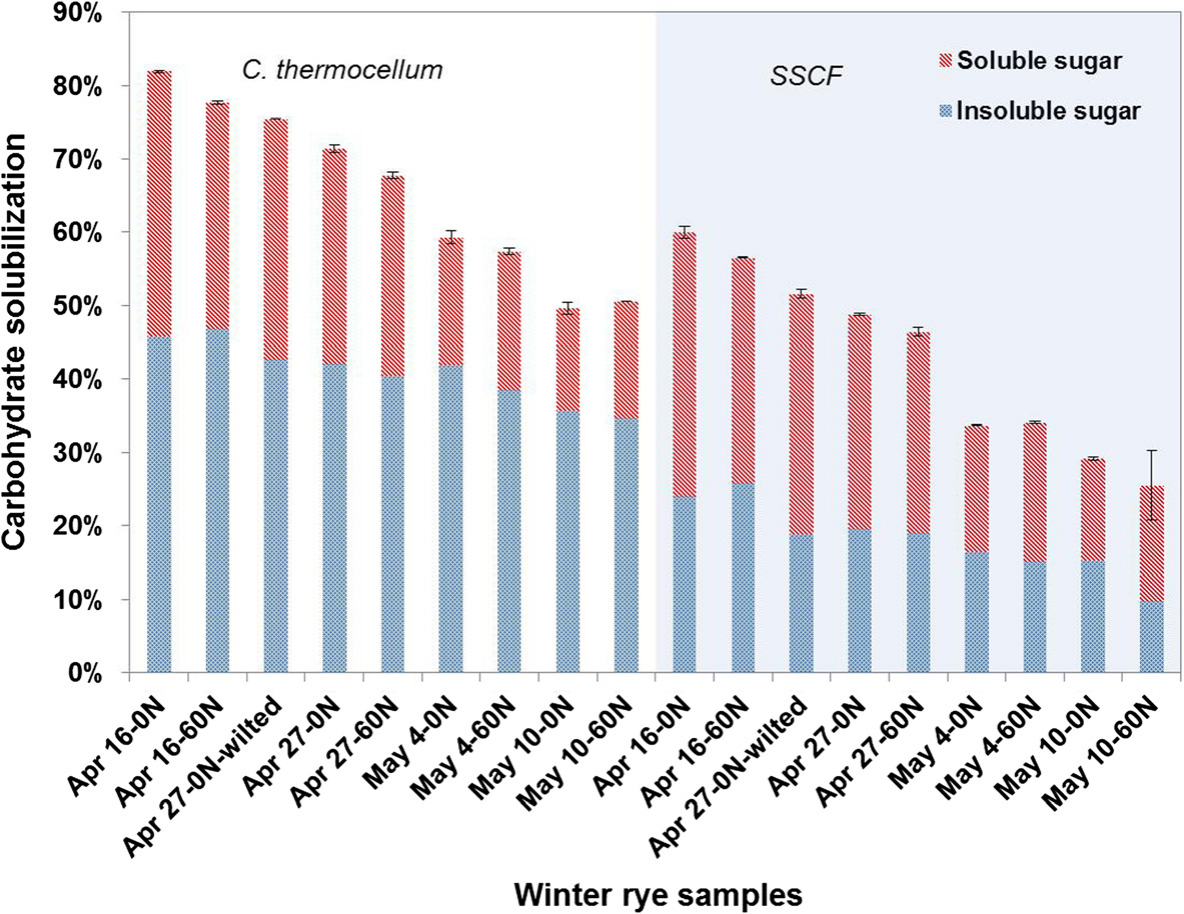
**Total carbohydrate solubilization by** ***C. thermocellum*** **fermentation and SSCF for various winter rye samples (error bars are from duplicate fermentations).**

Solubilization of winter rye was investigated at a low (approximately 10 g/L) carbohydrate loading rate (roughly 15 g/L total substrate) using two conversion systems: (1) fermentation by *C. thermocellum* and (2) simultaneous saccharification and cofermentation using commercial cellulase preparations (CTec2 and HTec2) and a xylose-fermenting strain of *Saccharomyces cerevisiae*. A comparison of total carbohydrate solubilization between SSCF and *C. thermocellum* fermentation is shown in Figure 1 (raw data in Additional file 1: Table S3). Contributions from the water soluble and insoluble fractions are also shown for each conversion system. The water soluble fraction was assumed to be solubilized 100%. The overall carbohydrate solubilization in the water insoluble fraction is shown in Additional file 1: Figure S1, and solubilization of individual carbohydrate components glucan, xylan, and arabinan can be found in Additional file 1: Figures S2, S3, and S4. Between the April 16 harvest date and the May 10 harvest date, the total carbohydrate solubilization dropped from 82% to around 50% for *C. thermocellum* fermentation while it dropped from about 60% to 30% for SSCF. When accounting for water insoluble fraction of biomass only, solubilization ranged from 72% to 42% over the sampling period for *C. thermocellum* and from 38% to 12% for SSCF (Additional file 1: Figure S1). *C. thermocellum* achieved much higher overall total carbohydrate solubilization (approximately 20% of the original carbohydrate present, corresponding to 30 to 50% greater solubilization) compared to SSCF, primarily because conversion of insoluble carbohydrate was approximately twice as high for *C. thermocellum* as compared to SSCF throughout the sampling period. Carbohydrate solubilization was slightly higher for the samples without nitrogen fertilization compared to the 60 kg N/ha fertilizer rate, likely because of the more advanced maturity of the fertilized plants. However, statistical analysis turned out that there were only nitrogen fertilization effects on the samples of April 16 and April 27 for *C. thermocellum*, and there were no nitrogen fertilization effects on all sample for SSCF.

**Figure 2:**
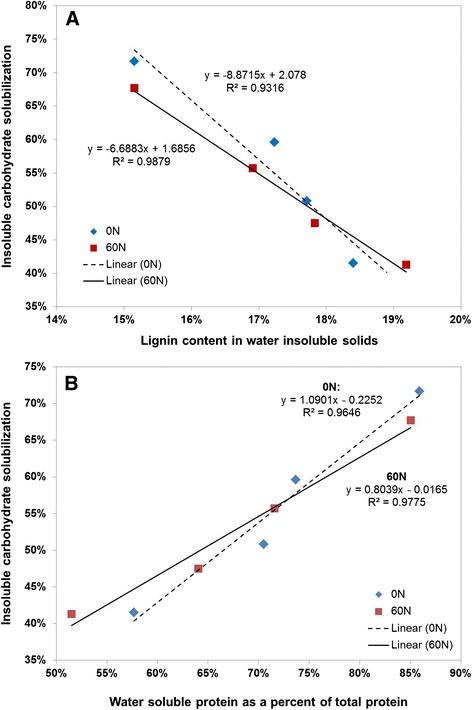
**Insoluble carbohydrate solubilization by** ***C. thermocellum*** **as a function of (A) lignin content of water insoluble solids and (B) water soluble protein as a percent of total protein.**

Carbohydrate solubilization was observed to decrease as winter rye matured. As shown in Figure 2A, carbohydrate solubilization was negatively correlated with increasing lignin content, but was positively correlated with the percent of protein that is soluble (Figure 2B). The recalcitrance of cellulosic biomass to biological attack is a complex and not fully understood phenomenon, with contributing factors including inaccessibility to enzymes and microbes, chemical linkages between carbohydrate and non-carbohydrate components, and unproductive binding of enzymes to lignin [22-24]. The greater effectiveness of *C. thermocellum* compared to SSCF using fungal cellulases is notable, not easily explained, and an interesting topic for future research. This difference is particularly puzzling since the cellulosome, thought to be primarily responsible for solubilization by *C. thermocellum* [25,26], has a molecular weight over an order of magnitude larger than the largest enzyme produced by fungal cellulases and thus might be expected to be less effective at accessing glycosidic bonds.

### Potential products and revenue

**Figure 3:**
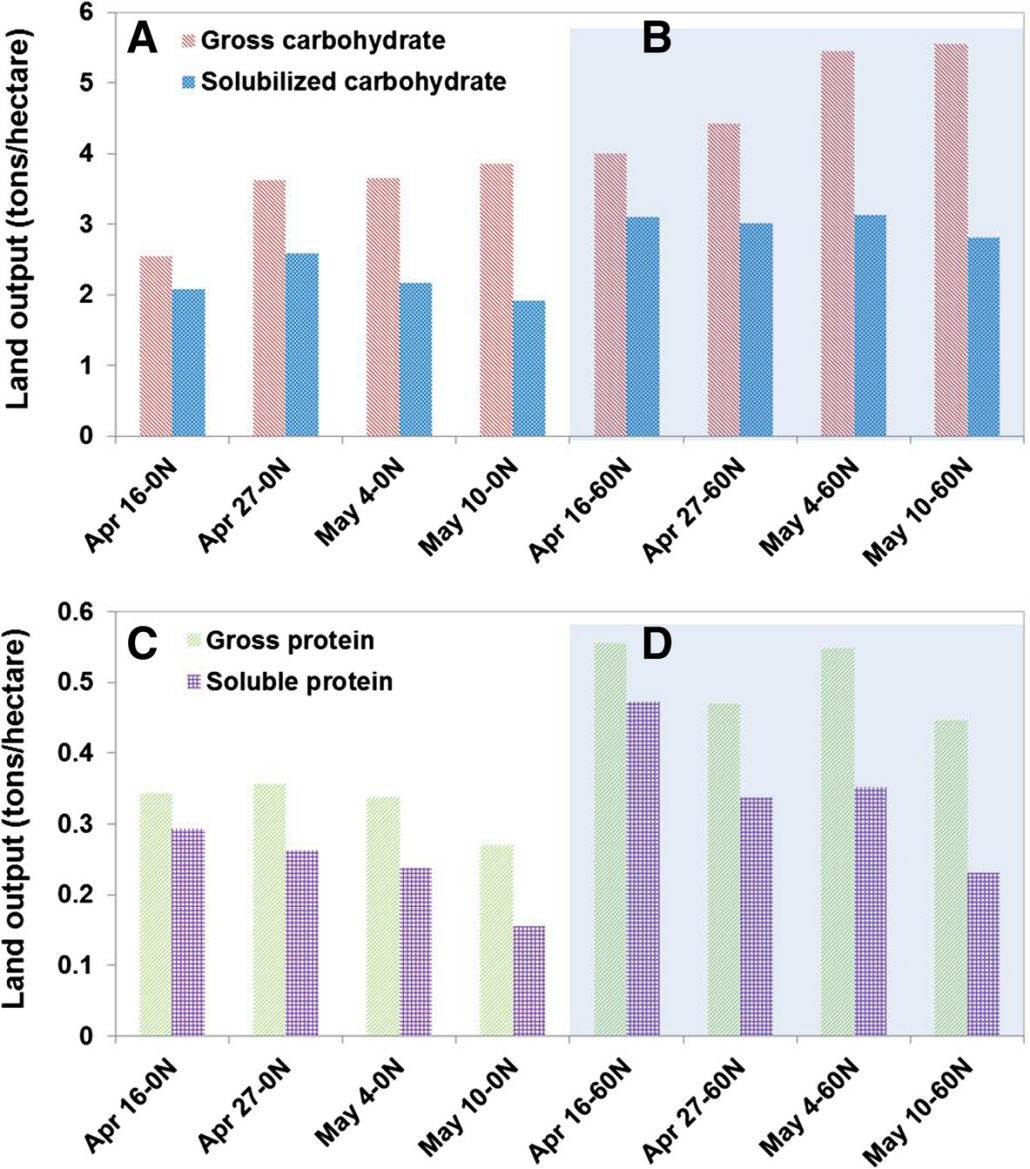
**Unit land output for gross carbohydrate, solubilized carbohydrate, gross protein, and soluble protein as a function of harvest time (A and C: no added nitrogen, B and D: 60 kg/ha nitrogen).**

As shown in Figure 3A, total carbohydrate harvested increases for later harvest dates while solubilized carbohydrate is quite flat. Gross protein is relatively flat while soluble protein has a decreasing trend as winter rye matures (Figure 3B). For either carbohydrate or protein, adding nitrogen fertilization significantly increased the system output (up to about 50% increase).

**Figure 4:**
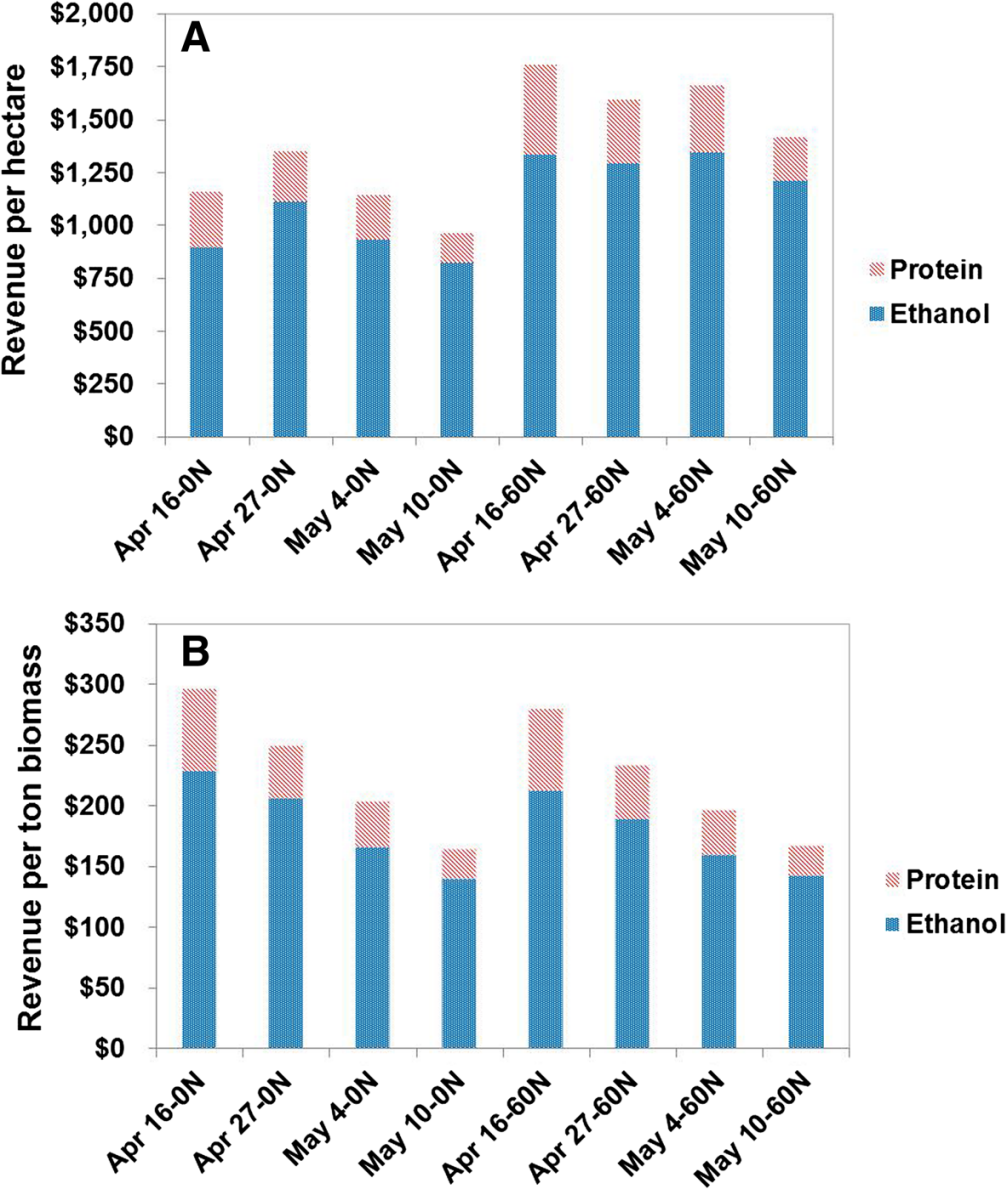
**Revenue potential on the basis of unit land area (A) or unit biomass output (B) as a function of harvest time (assumed ethanol price: $0.66/L, protein price: $900/t).**

We calculate potential revenue for winter rye as a bioenergy feedstock by assuming that the solubilized carbohydrate will be converted into ethanol and sold at a price of $0.66/L and that the soluble protein can be recovered for feed protein and sold for $900/t. This revenue can be expressed in terms of unit land area or unit biomass weight as shown in Figure 4, and in either case ethanol represents most of the revenue. On a per-hectare basis (of particular relevance to a farmer with a fixed land area), the potential revenue is relatively constant through most of the sampling period but falls off at the last harvest date (Figure 4A). Thus, farmers might have flexibility to choose harvesting dates for winter rye within a several week window depending on the weather and timing for planting the summer crop. As with summer annual crops, adding nitrogen fertilizer to poor soil significantly increases potential revenue by an amount that will often more than cover the cost of fertilization. Decreasing protein content and decreasing digestibility are reflected in potential revenue per ton dry feedstock decreasing as winter rye matures (Figure 4B). This implies that the price for winter rye per unit weight will likely drop as it mature, even as more biomass is produced.

Increasing costs of protein feeds for livestock is a concern for USDA [27]. Protein from winter rye as a winter crop can potentially add significantly to US feed production. With typical values of 44.5 bu/acre, 60 lb/bu, and 40% protein content for soybean, the protein output is 1.2 ton/ha. Based on the soluble protein values in Figure 3B, a winter rye second crop would increase protein output per unit land by 0.25 to 0.4 ton/ha or about 20% to 35%. However, the feed value of recovered winter rye protein for dairy, cattle, swine, and poultry also needs to be evaluated. Although it has been demonstrated that differences in cultivars are far less significant than differences in maturity, there is likely some yield gain to be made from a study across cultivars that includes data from a range of growth stages, as varieties traditionally used for soft dough stage harvest may not be those best suited for early harvest [12].

## Conclusions

As a winter crop, winter rye can provide significant lignocellulosic feedstock for biofuel production per unit land area before planting main summer crop. Winter rye changes composition and biological reactivity rapidly in the spring. Immature winter rye is more amenable for *C. thermocellum* fermentation than SSCF (approximately 20% difference). Planting winter rye as winter crop can add significantly to farmer’s income. If pricing was based on quality, as it is for forage, potential revenue would be quite stable for different harvest dates, which offers flexible timing for harvesting. Biofuel production using winter rye as feedstock could provide significant feed protein as coproduct.

## Methods

### Winter rye planting and harvesting

The cultivar Aroostook rye was planted at a density of 150 lbs/acre on 19 September 2011 in Rock Springs, PA on a Hagerstown silty clay loam soil. For the samples with fertilization (ammonium sulfate, 21-0-0), 60 kg nitrogen per hectare (kg/ha) were hand applied in the fall at planting. Three field replicates of one half square meters each were hand harvested leaving two inches stubble. Yields were estimated on a dry weight basis after drying in a 105°C oven until the mass was constant (24 to 28 h). The plant material was air dried indoors on racks until the moisture level stabilized (3 or 4 days), with the exception of one large non-fertilized plot that was harvested mechanically on April 27, wilted in the field for a 24-h period, then air dried indoors without racks but with periodic turning. A description of the biomass samples characterized and then fermented is provided in Table 1. Samples were taken on each harvesting date from both 0 and 60 kg/ha fertilization plots. Samples used in this study were milled to pass through a 0.5 mm screen using the RETSCH ultra-centrifugal mill ZM 200.

### Strains, enzymes, and culturing media

A xylose utilizing *S. cerevisiae* strain (Mascoma Corporation, Lebanon, NH, USA) prepared in YPD media (Sigma Y1375, St. Louis, MO, USA) was used for SSCF inoculation. The KN medium, developed by Kadam and Newman [28] and consisting of 0.3% (*v/v*) corn steep liquor supplemented by 5 mM MgSO_4_, was used in all SSCF experiments. Cellic CTec2 and HTec2 were kindly provided by Novozymes (Franklinton, NC, USA). *C. thermocellum* DSM 1313 was from DSMZ (Braunschweig, Germany). Chemically-defined media for thermophilic clostridia (MTC), with components in solutions A (carbohydrate), B (citrate and bicarbonate buffer), C (nitrogen source), D (minerals and reducing agent), E (vitamins), and F (supplemental MOPS buffer), was prepared according to Shao et al. [29] with the exception that solution A contained winter rye sample as substrate. All chemicals were reagent grade and were obtained from Sigma (St. Louis, MO, USA), unless indicated otherwise.

### SSCF

A 0.75 g winter rye sample was added into 125-ml serum bottles and supplemented with 41 ml DI water and 0.2 g CaCO_3_. The bottles were crimp-sealed, purged with N_2_, and sterilized by autoclaving at 121°C for 35 min. After cooling, a 5 ml filter-sterilized solution consisting of 0.15 ml corn-steep liquor, 0.03 g MgSO4, and 4.85 ml DI water was added by syringe. The bottles were then injected with 2 ml filter-sterilized enzyme solution consisting 0.0425 ml cellulase complex Cellic CTec2 (10 mg protein/g total solids), 0.0209 ml endoxylanase Cellic HTec2 (5 mg protein/g total solids), and 1.936 ml DI water. Finally, 2 ml yeast inocula prepared in YPD media at 35°C was injected. The bottles were placed in a shaking incubator (New Brunswick Scientific, Innova 4080, Enfield, CT, USA) with temperature controlled at 35°C and rotation speed set at 200 rpm. After incubating for 120 h, the content of an entire bottle was collected and centrifuged. The supernatant was discarded after sampling for HPLC measurement. The pellets were resuspended to 50 mL with DI water and centrifuged again. The resulting pellets were analyzed for residual glucan, xylan, and arabinan to calculate carbohydrate solubilization.

### *C. thermocellum* fermentation

Winter rye was ground so that the particles could pass through a 0.5 mm screen. A sample of 0.75 g was added into 125-ml serum bottles and supplemented with 35 ml DI water. The bottles were crimp-sealed, purged with N_2_, and sterilized by autoclaving at 121°C for 35 min. After that, 2 ml B, 1 ml C, 1 ml D, 1 ml E, and 5 ml F stock solutions were added by syringe. The bottles were then injected with 5 ml inocula from an exponential phase culture grown on 5 g/L Avicel PH 105. The bottles were placed in a shaking incubator (New Brunswick Scientific, Innova 4080, Enfield, CT, USA) with temperature controlled at 55°C and rotation speed set at 200 rpm. Sample collection and processing were the same as described for SSCF.

### Analytical methods

Water soluble fraction of winter rye samples was determined by measuring weight loss after incubating 15 g/L sample at 55°C in a shaking incubator for 1 h followed by washing the remaining solids three times with the same amount of water. The carbohydrate content in the water insoluble fraction of winter rye samples and residual pellets collected and freeze-dried after SSCF and *C. thermocellum* fermentation were determined by quantitative saccharification [30], with biomass quantities scaled-down to one third. For carbohydrate content in the water soluble fraction of winter rye samples, dilute acid hydrolysis was performed by adding 0.125 ml 72% (wt) H_2_SO_4_ to 28.725 ml supernatant and autoclaving at 121°C for 1 h. Product concentrations were obtained using a Waters HPLC system (#2695, Milford, MA, USA) with an Aminex HPX-87H column (Bio-rad, Hercules, CA) operated at 60°C and an RI detector. A mobile phase of 5 mM H_2_SO_4_ was used at a flow rate of 0.6 mL/min. Carbohydrate solubilization was calculated as a percentage of originally-present glucan, xylan, or arabinan solubilized, based on analysis of residual solids. Protein content in the winter rye samples was calculated using a factor of 6.25 from nitrogen content determined by combustion approach on a Shimadzu TOC/TON analyzer (TOC-V_CPH_ and TNM-1).

## Additional file

Additional file 1:
**Supplemental material.** Table S1. Composition of water soluble fraction of winter rye samples. Table S2. Composition of water insoluble fraction of winter rye samples. Figure S1. Overall carbohydrate solubilization of the water insoluble fraction. Figure S2. Glucan solubilization of the water insoluble fraction. Figure S3. Xylan solubilization of the water insoluble fraction. Figure S4. Arabinan solubilization of the water insoluble fraction. Table 1. Production data for winter rye samples.

## Abbreviations

ADF: acid detergent fiber
CBP: consolidated bioprocessing
CP: crude protein
DOM: digestible organic matter
IVTD: *in vitro* true digestibility
NDF: neutral detergent fiber
SHP: separate hydrolysis and fermentation
SSCF: simultaneous saccharification and co-fermentation
SSF: simultaneous saccharification and fermentation
