# Supplementary material for: Winter rye as a bioenergy feedstock: impact of crop maturity on composition, biological solubilization and potential revenue
Source: Biotechnol Biofuels. 2015 Feb 27;8:35. doi: 10.1186/s13068-015-0225-z (PMC4367844; doi:10.1186/s13068-015-0225-z)
Supplement: Additional file 1: — Supplemental material. Table S1. Composition of water soluble fraction of winter rye samples. Table S2. Composition of water insoluble fraction of winter rye samples. Figure S1. Overall carbohydrate solubilization of the water insoluble fraction. Figure S2. Glucan solubilization of the water insoluble fraction. Figure S3. Xylan solubilization of the water insoluble fraction. Figure S4. Arabinan solubilization of the water insoluble fraction. Table 1. Production data for winter rye samples. [file 13068_2015_225_MOESM1_ESM.docx]

# Supplemental material

**Table S1**. Composition of water soluble fraction of winter rye samples.

|  | **Glucose** | **Fructose** | **Arabinose** | **Protein** | **Mass bal** |
| --- | --- | --- | --- | --- | --- |
| Apr 16-0N | 18.4% | 45.1% | 0.6% | 20.6% | 84.6% |
| Apr 16-60N | 18.8% | 40.4% | 0.5% | 22.9% | 82.6% |
| Apr 27-0N-wilted | 22.1% | 48.7% | 0.6% | 17.4% | 88.8% |
| Apr 27-0N | 22.0% | 46.0% | 0.5% | 17.0% | 85.5% |
| Apr 27-60N | 21.5% | 41.5% | 0.4% | 17.6% | 81.0% |
| May 4-0N | 18.9% | 38.6% | 0.6% | 21.9% | 80.0% |
| May 4-60N | 19.0% | 39.3% | 0.6% | 20.1% | 79.0% |
| May 10-0N | 19.4% | 40.1% | 0.5% | 17.4% | 77.4% |
| May 10-60N | 20.8% | 41.5% | 0.4% | 16.4% | 79.2% |

**Table S2**. Composition of water insoluble fraction of winter rye samples.

|  | **Glucan** | **Xylan** | **Arabinan** | **Acetyl** | **Lignin** | **Protein** | **Ash** | **Mass bal** |
| --- | --- | --- | --- | --- | --- | --- | --- | --- |
| Apr 16-0N | 34.3% | 27.2% | 3.7% | 3.4% | 14.4% | 1.9% | 0.8% | 85.8% |
| Apr 16-60N | 35.4% | 26.7% | 3.5% | 3.6% | 16.5% | 2.0% | 0.8% | 88.4% |
| Apr 27-0N-wilted | 34.4% | 25.4% | 3.0% | 3.4% | 17.2% | 1.9% | 1.2% | 86.6% |
| Apr 27-0N | 37.1% | 26.3% | 3.0% | 3.7% | 16.5% | 2.4% | 0.8% | 89.7% |
| Apr 27-60N | 36.6% | 25.6% | 2.9% | 3.6% | 17.3% | 2.7% | 0.8% | 89.6% |
| May 4-0N | 37.7% | 25.9% | 2.8% | 3.7% | 18.1% | 2.2% | 0.8% | 91.1% |
| May 4-60N | 37.7% | 25.3% | 2.8% | 3.5% | 18.0% | 2.9% | 0.8% | 90.9% |
| May 10-0N | 38.8% | 25.1% | 2.4% | 3.5% | 16.3% | 2.3% | 0.8% | 89.3% |
| May 10-60N | 38.7% | 24.7% | 2.5% | 3.4% | 17.1% | 3.1% | 0.7% | 90.1% |

**Table S3**. Raw data carbohydrate solubilization.

|  | **Inititial weight, g** | **Moisture content** | **C. therm residual Carbohydrate, g** | | | **SSCF residual Carbohydrate, g** | | | |
| --- | --- | --- | --- | --- | --- | --- | --- | --- | --- |
|  |  |  | **Glucan** | **Xylan** | **Arabinan** | **Glucan** | **Xylan** | | **Arabinan** |
| Apr 16-0N | 0.75 | 8.7% | 0.042 | 0.036 | 0.0024 | 0.092 | 0.080 | | 0.016 |
| Apr 16-60N | 0.75 | 7.3% | 0.053 | 0.043 | 0.0029 | 0.103 | 0.083 | | 0.016 |
| Apr 27-0N-wilted | 0.75 | 6.5% | 0.061 | 0.048 | 0.0033 | 0.122 | 0.094 | | 0.015 |
| Apr 27-0N | 0.75 | 7.0% | 0.072 | 0.055 | 0.0038 | 0.129 | 0.098 | | 0.015 |
| Apr 27-60N | 0.75 | 7.0% | 0.082 | 0.059 | 0.0041 | 0.135 | 0.100 | | 0.015 |
| May 4-0N | 0.75 | 7.8% | 0.104 | 0.073 | 0.0051 | 0.169 | 0.119 | | 0.015 |
| May 4-60N | 0.75 | 7.5% | 0.109 | 0.076 | 0.0055 | 0.168 | 0.117 | | 0.015 |
| May 10-0N | 0.75 | 7.6% | 0.134 | 0.088 | 0.0063 | 0.187 | 0.124 | 0.014 | |
| May 10-60N | 0.75 | 7.2% | 0.133 | 0.086 | 0.0064 | 0.198 | 0.131 | 0.014 | |


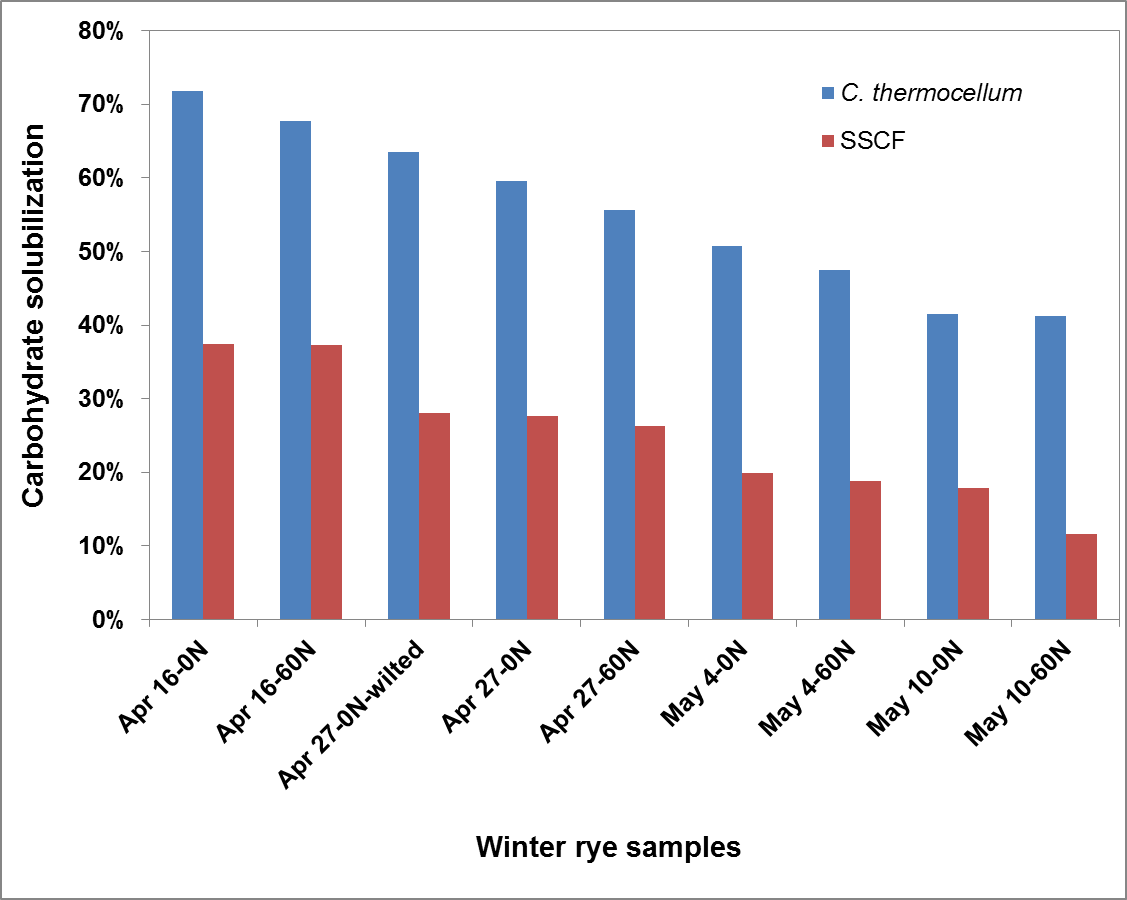


**Figure S1**. Overall carbohydrate solubilization of the water insoluble fraction.


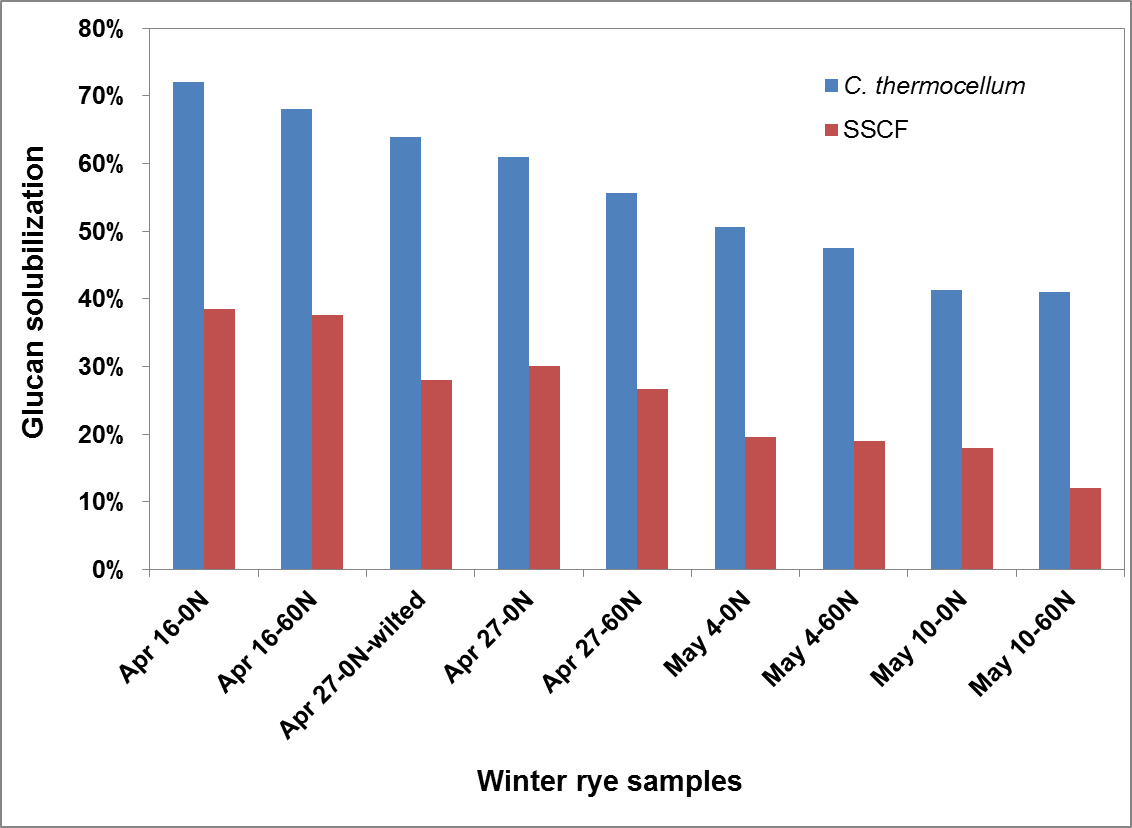


**Figure S2**. Glucan solubilization of the water insoluble fraction.


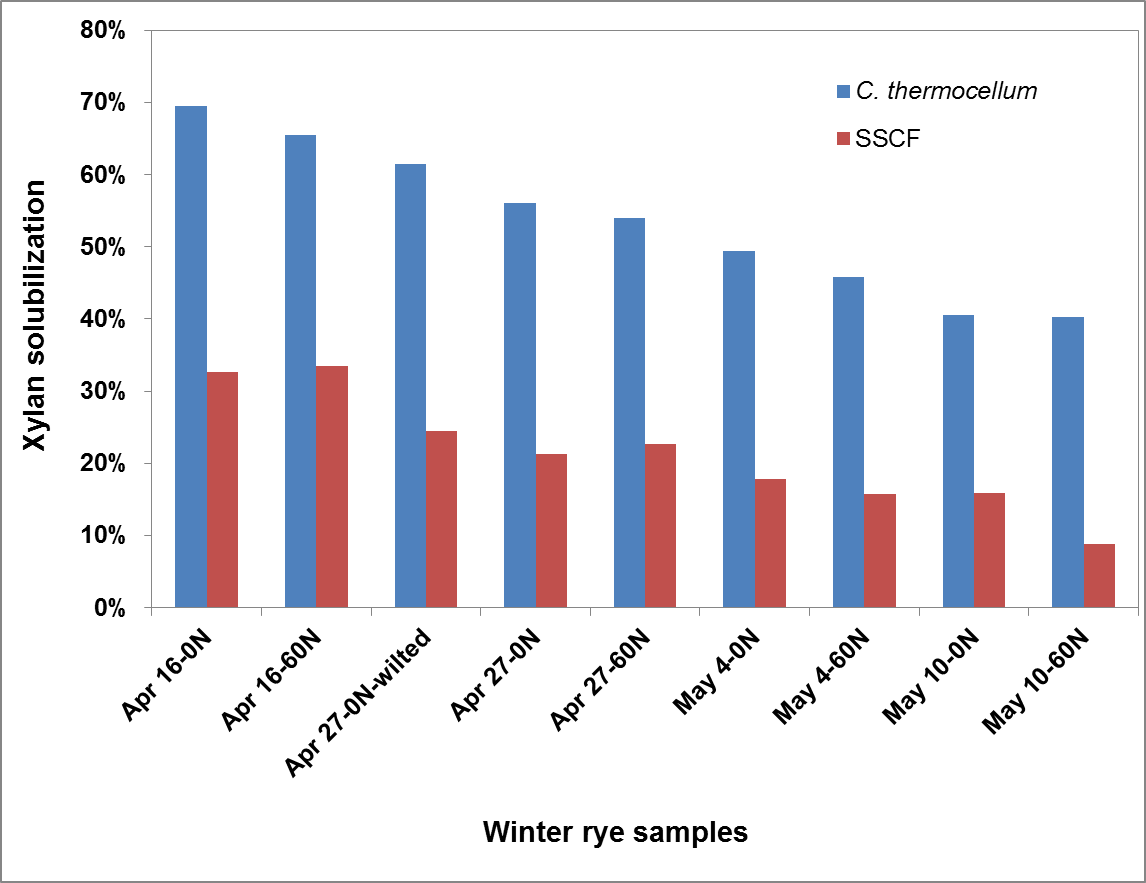


**Figure S3**. Xylan solubilization of the water insoluble fraction.


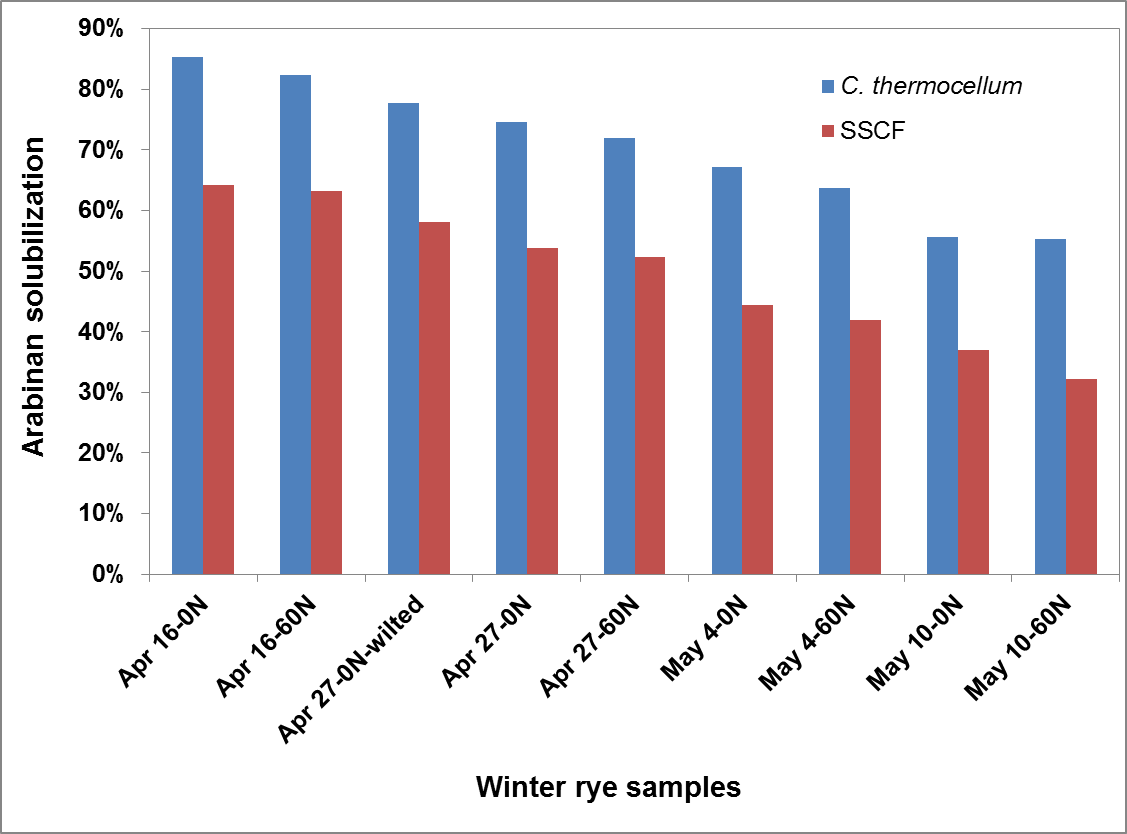


**Figure S4**. Arabinan solubilization of the water insoluble fraction.
